# Supplementary figures and images for: The Effect of Particle Size and Surface Roughness of Spray-Dried Bosentan Microparticles on Aerodynamic Performance for Dry Powder Inhalation
Source: Pharmaceutics. 2020 Aug 13;12(8):765. doi: 10.3390/pharmaceutics12080765 (PMC7465523; doi:10.3390/pharmaceutics12080765)

## Slide 1
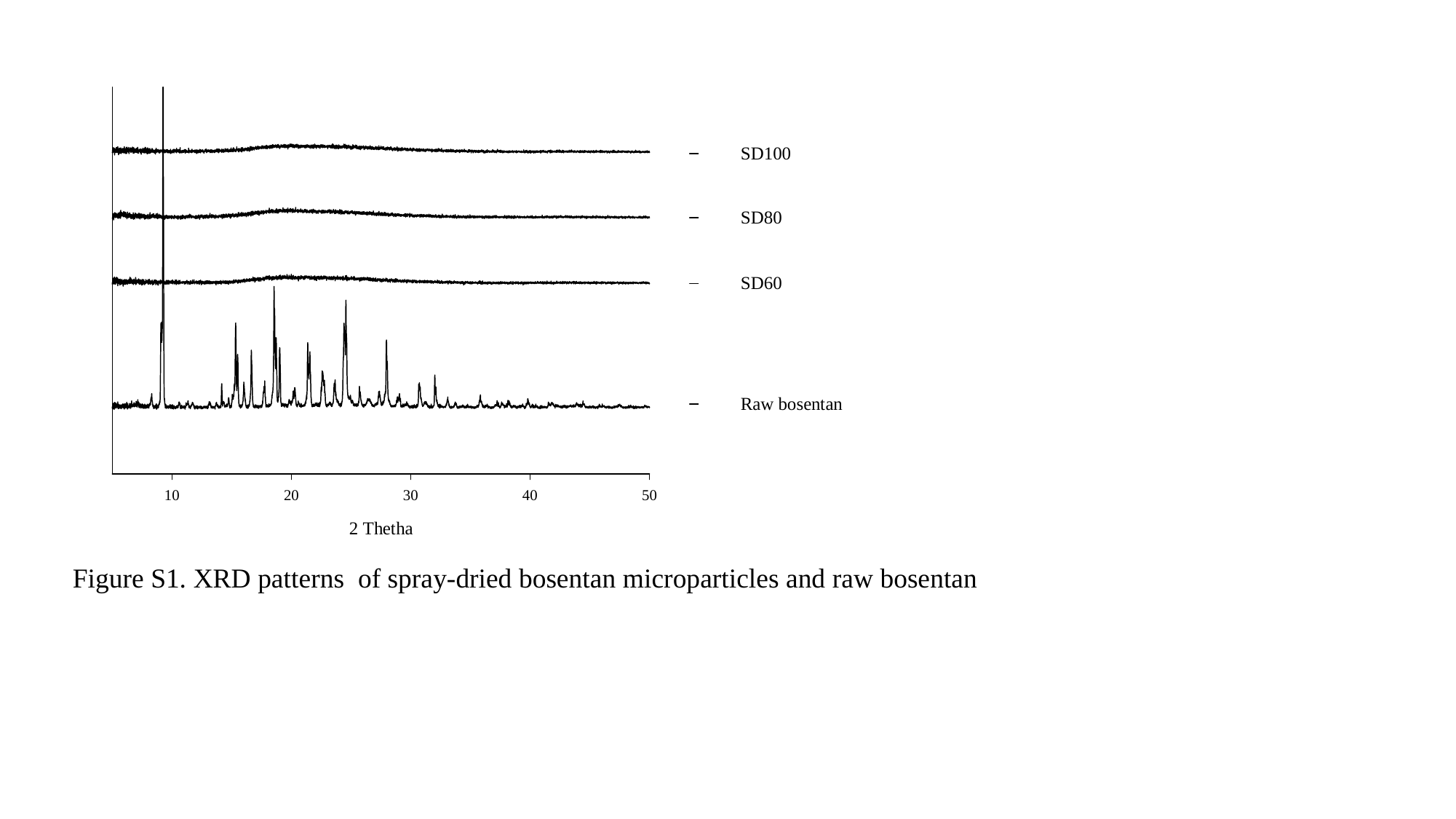

Figure S1. XRD patterns of spray-dried bosentan microparticles and raw bosentan

Supplement: Supplementary file 1 [file pharmaceutics-12-00765-s001.zip › Supplementary material. Figure S1.pptx]
